# The Validity and Reliability of the Force Plates and the Linear Position Transducer in Measuring Countermovement Depth and Velocity During Countermovement Jump

**DOI:** 10.3390/s25216542

**Published:** 2025-10-23

**Authors:** Zheng’ao Li, Wenyue Ma, Ling Zhang, Wenfei Zhu, Qian Xie, Yuliang Sun

**Affiliations:** School of Physical Education, Shaanxi Normal University, Xi’an 710119, China; lizhengao@snnu.edu.cn (Z.L.); mawnyue@snnu.edu.cn (W.M.); zhangling1@snnu.edu.cn (L.Z.); wzhu@snnu.edu.cn (W.Z.)

**Keywords:** vertical jump, center of mass, performance monitoring

## Abstract

Countermovement jump (CMJ) is a key test for evaluating lower-limb neuromuscular function, and accurate measurement of countermovement depth (CMD) and countermovement velocity (CMV) is critical for determining optimal performance. However, the measurement validity and reliability of CMD and CMV—particularly when obtained from force plates (FP) and linear position transducers (LPT)—have remained uncertain. This study determined the validity and reliability of FP and LPT for measuring CMD and CMV. Twenty-eight male recreational athletes performed the CMJ test, and the variables were synchronously acquired by Motion Capture (MC), FP, and LPT. The test was divided into two sessions, with participants completing three maximal effort CMJs per session, and the second session occurred more than 48 h after the first. The reliability was evaluated using the intraclass correlation coefficient (ICC), and the validity was evaluated with linear Pearson’s correlation coefficient (r), one-way ANOVA with repeated measures, and Bland–Altman plots. The reliability results for FP and LPT indicated good to excellent (ICC = 0.809–0.900). Compared with MC, the FP showed a high to very high correlation (r = 0.894–0.937), and the LPT showed a high correlation (r = 0.721–0.726). When precise quantification of CMD/CMV is required, FP should be preferred. If only an LPT is available, it is best used for within-athlete longitudinal monitoring with a consistent setup, and cross-device comparisons should be avoided.

## 1. Introduction

The countermovement jump (CMJ) is arguably the most commonly used test for monitoring neuromuscular status and distinguishing injury rehabilitation in the lower-limb [1,2,3]. Recent studies have increasingly focused on the countermovement of depth (CMD) and velocity (CMV) as a key methodological factor [4,5,6] during the CMJ, and the CMD and CMV influence the force-time curve of the CMJ, further affecting these important evaluation metrics (such as jump height, modified reactive-strength index) [5,6,7]. However, the CMD and CMV are influenced by participants’ execution strategies (i.e., self-habits and verbal cues) [8,9], and they were often ignored in CMJ tests and not reported in many study results [3]. Therefore, this lack of reporting may be a significant source of error when comparing metrics within or between individuals [6]. It is necessary to report the depth and velocity of countermovement, which allows practitioners to better understand changes in participants’ neuromuscular functioning [10].

Various instruments can acquire the raw signals required to compute CMJ outcome variables during the CMJ test, including motion capture systems, force plates, linear position transducers, jump mats, and simplified optical measurement systems [11,12,13,14,15,16,17]. However, some devices (e.g., jump mats and simplified optical measurement systems) are restricted to the flight times (FT) method, owing to the lack of position and force-time data, so that they cannot calculate CMD and CMV [18]. As the ‘gold standard’ methods, the motion capture systems calculate the position and velocity of the centre of mass (COM) by tracking the reflective markers placed on the total body bony landmarks [19,20], but they are expensive, impractical, and are restrictedly used in the laboratory. Based on the FP, the Double Integration (DI) Method is one of the most reliable and accurate approaches to evaluate the displacement of COM (e.g., JH and CMD) [7,20,21,22]. The difference between the jump height calculated based on the DI method and that measured by the motion capture system is negligible [23] when the sampling frequency of the FP is equal to or larger than 1000 Hz [21]. Additionally, as a portable and practical instrument, the LPT quantifies an object’s displacement utilising optical encoding technology by detecting positional changes in a tethered cable and converting these into metric measurements [13]. Although some studies report that LPTs overestimate jump height relative to force plates or motion capture [12,13,14], LPTs provide acceptable reliability and validity for field-based monitoring and, given their portability, offer a lower-cost alternative when MC/FP are impractical [12,13,24,25,26]. However, few studies have focused on the reliability of CMD and CMV measured by different devices [27], and the validity and reliability of the depth and velocity of countermovement by FP and LPT are unclear.

Therefore, this study aimed to determine the concurrent validity of depth and velocity of countermovement measured by the force plates (FP) and the linear position transducer (LPT) by comparing with motion capture (MC). Meanwhile, the reliability of depth and velocity of countermovement, calculated based on FP and LPT, was determined by inter-day retesting. The first hypothesis of this study was that force plates (FP) would demonstrate excellent validity for measuring countermovement depth (CMD) and velocity (CMV), showing strong agreement with the motion capture (MC). In contrast, the linear position transducer (LPT) was hypothesised to exhibit an overestimation of CMD and CMV compared to other devices. The second hypothesis was that both the FP and the LPT would demonstrate good to excellent between-day reliability in their measurements of CMD and CMV.

## 2. Methods

### 2.1. Participants

Twenty-eight male recreational athletes (height 180.1 ± 6.7 cm, body mass 72.1 ± 9.3 kg, age 21.7 ± 2.4 years) with various sporting histories, including athletics, basketball, and powerlifting, were included. The participants were uninjured in the past six months, willingly chose to participate in the study, and supplied written consent. The study followed the ethics guidelines and principles of the Declaration of Helsinki. It received approval from the Ethics Committee at Shaanxi Normal University (Approval No. 202416044, October 2024).

### 2.2. Study Design

The experiment was conducted in the Biomechanics Laboratory of Shaanxi Normal University. All athletes completed one familiarisation session before the experiment. Before the experiment began, the players warmed up with 5 min of jogging and 10 sub-maximal jumps. A 10-camera infrared motion capture system (Oqus700+, Qualisys AB, Gothenburg, Sweden) was employed to record kinematic data at a sampling frequency of 200 Hz. The Qualisys motion-capture system was calibrated at the start of each session using the vendor L-frame to define the global coordinate system and a dynamic wand procedure; the final volume calibration residual was ≤0.5 mm. Kinetic data were collected using two force plates (Kistler 9260AA6, 0.5 m × 0.6 m; Kistler Instruments Corp., Winterthur, Switzerland) with a sampling frequency of 1000 Hz. Kistler force plates were zeroed and checked with certified 5 kg masses placed centrally; measured vertical forces matched the expected loads after gravity correction. Motion capture data were collected synchronously with ground reaction force data (Qualisys Track Manager, Qualisys). The motion capture and force plates were hardware-synchronized within the Qualisys acquisition environment via BNC cabling and a common TTL trigger. Participants wore uniforms and tight pants, were given a pair of shoes, and used 61 reflective markers placed on bony landmarks. The sticking model referred to the modified Helen Hayes model [28,29] (Appendix A). At the same time, participants were attached to the LPT (GymAware, Kinetic Performance Technology, Canberra, Australia) via a waist belt. This configuration approximates the waist-point (pelvic) method, using belt/pelvis motion as a surrogate for the whole-body center of mass. Previous studies have shown that pelvis-based kinematics can approximate COM motion during jumping tasks within acceptable error bounds [30,31]. The LPT was wirelessly connected to an iPad running the proprietary GymAware software (v2.4.1). To minimise systematic measurement error, the position of the LPT transducer was standardised across all participants and testing sessions [13]. Before each measurement, the tether was fully retracted, and the device was “zeroed” using the software’s calibration function to establish a consistent zero baseline. The GymAware LPT employs an adaptive sampling rate (50 Hz) and digitally filters the raw signal to minimise noise. Subsequently, the processed tether length data is converted into vertical displacement by applying trigonometric corrections that account for any horizontal movement of the tether [32].

The LPT (e.g., GymAware) is widely recognized as a reliable tool for measuring velocity and displacement in humans during jumping [12,13], and the tether force it exerts is considered negligible with respect to force plate measurements. However, to ensure scientific rigor, participants stood upright on a force plate for 9 s under two conditions (with and without the LPT), with vGRF recorded continuously. The mean force from the middle 3 s was compared between conditions to assess the potential influence of the LPT tether.

**Figure 1:**
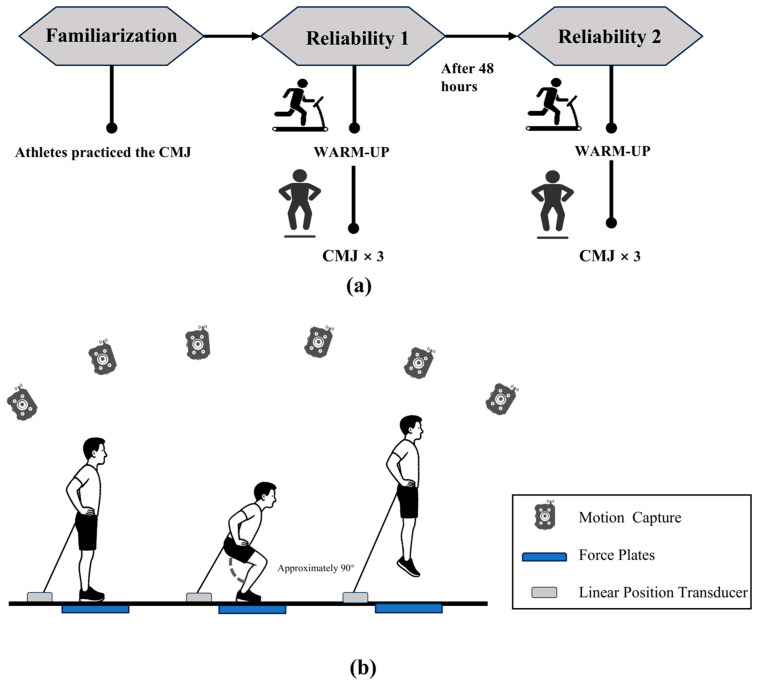
(**a**) Schematic representation of the study design. (**b**) Experimental environment and setup.

After the FP were tared, participants stood quietly on the force plates for ≥1 s before performing the CMJ, and the distance between their feet should equal shoulder width. And receiving a “3, 2, 1, jump” instruction, the counter movement was performed at full speed until the knee angles were flexed (approximately) 90° [33], jumping with maximum effort. Participants completed a brief familiarization session with practice CMJs before data collection. A standardized setup and verbal cues were used (shoulder-width stance, hands on hips, no pause during the countermovement, and knee joint flexion control) to enhance execution consistency. Sixty seconds of rest were given between each trial to ensure proper recovery and sustain optimal jump performance. After 48 h, participants completed a second CMJ test while wearing the same tight pants and shoes (Figure 1). The protocol for the CMJ assessments was identical for both tests to assess the test–retest variable reliability of different devices.

### 2.3. Data Processing

Countermovement depth and velocity of LPT were calculated based on changes in displacement and time from the starting position (standing upright) to the lowest point of displacement, and recorded using the manufacturer’s software (GymAware v2.4.1, GymAware, Kinetic Performance Technology, Canberra, Australia). Pre-processed kinematic and kinetic data (C3D format) were imported into Visual3D (V6.0, C-Motion, Germantown, MD, USA). A fourth-order Butterworth low-pass digital filter was used to filter the data, with kinematic and kinetic cutoff frequencies set at 14 Hz and 50 Hz [34,35].

**Figure 2:**
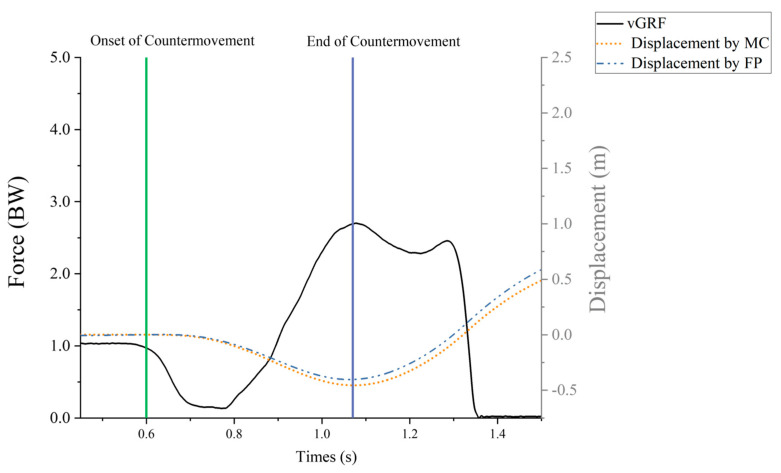
A representative example of a countermovement jump trial and the occurrence of key events. Note: The green line is the moment of the start of the reverse maneuver, where the vGRF is 20 N smaller than the body weight; the purple line is the lowest point of the center of mass.

The COM variables were used in the rigid body kinematic model to compute the whole-body relative to the laboratory coordinate system. Start and end events were created in V3D with the threshold set to 20 N [36]. The start event is when the vertical ground reaction force (vGRF) drops below one’s body weight minus 20 N, and the end event is when the vGRF falls below 20 N. Find the moment of the lowest vertical displacement of COM within the start and end event times, from the beginning of the action to the lowest vertical displacement as time to the lowest COM. The change in COM minimum displacement and the time to lowest COM required determined MC’s countermovement depth and velocity. The kinetics data were exported into MATLAB R2023b (MathWorks), and the vertical combined forces from the two force plates were used in a custom script to calculate COM vertical displacements based on the double integrals (DI) of acceleration (net force divided by body mass). After filtering, the net vertical force data were double integrated to calculate the vertical displacement of the center of mass (COM). To reduce integration drift, we applied start constraints at movement onset—defined as the instant when the vertical ground reaction force fell below body weight minus 20 N—and set both the COM velocity and displacement to zero at that time. In this formulation, Equation (1), t_0_ represents the start time of the movement. The net force F*_net_*(*τ*) is the difference between the measured vertical ground reaction force F*_vGRF_*(*τ*) and the product of body mass m and gravitational acceleration g. The variable τ serves as the variable of integration throughout the calculation process. Figure 2 provides a visual representation of the CMJ with the associated phases of the force-time curve. Additionally, countermovement velocity was calculated as the change in COM vertical position from movement onset (t0) to the end of the countermovement (minimum COM), divided by the elapsed time (t minus t0). Values of CMD were reported as positive magnitudes.

(1) $$s\left(t\right)=\iint_{t_{0}}^{t}\frac{F_{\mathrm{net}}\left(\tau \right)}{m}d\tau d\tau$$

### 2.4. Statistical Analysis

All data are presented as mean ± standard deviation (SD), and the normality was checked with the Shapiro–Wilk test. A one-way ANOVA with repeated measures with Holm–Bonferroni post hoc corrections for different variables was used to compare countermovement depth and velocity with the various devices. Where the sphericity assumption was violated, the Greenhouse–Geisser-corrected *p*-values in the results were reported.

Comparison of different devices was achieved using Pearson product-moment correlation analysis (r). The motion capture system is the “gold standard”, so the MC variable is the dependent variable. While correlation analyses show how two variables are related, they do not necessarily reveal how much the values agree or disagree. Therefore, the Bland–Altman plots and 95% limits of agreement (LoA) were used to demonstrate the bias between the MC, the FP, and LPT to show the agreement levels. The magnitude of correlation between the LPT and the MC, the FP and the MC was assessed using the following thresholds: between 0.5 and 0.69 was considered moderate, between 0.70 and 0.89 was considered high, and over 0.9 was considered very high [26]. Additionally, we computed derived absolute and squared errors for FP and LPT. The mean absolute error (MAE) was the mean of the absolute differences; the root-mean-square error (RMSE) was the square root of the mean of the squared differences. Normalized RMSE (NRMSE, %) was defined as NRMSE = 100 × RMSE / mean (MC), reported separately for CMD and CMV.

All trials based on the average of countermovement depth and velocity for different devices on each testing day were used for inter-day reliability analysis. Reliability was evaluated by calculating the coefficient of variation (representing the typical error and expressed as a CV) and the intraclass correlation coefficient (ICC) with 95% confidence intervals (95% CI) using an Excel (Office 2021) spreadsheet. Day-to-day reliability was evaluated with intraclass correlation coefficients using a two-way random-effects model, absolute agreement, average measures. Values of ICC less than 0.5 are indicative of poor reliability, values between 0.5 and 0.75 indicate moderate reliability, values between 0.75 and 0.9 indicate good reliability, and values greater than 0.90 indicate excellent reliability [37]. The minimal detectable change (MDC) was determined as a statistical estimate of the smallest amount that can be considered a real change. Vertical ground reaction forces with and without the LPT were compared using a two-tailed paired t-test (α = 0.05). Data are reported as mean ± SD. The effect size (ES) was calculated as Cohen′s d. The magnitude of effect sizes was categorized as none (0 < Cohen′s d < 0.20), small (0.20 < Cohen′s d < 0.50), medium (0.50 < Cohen′s d < 0.80), and large (Cohen′s d ≥ 0.80). This study employed SPSS 27.0 (Statistical Package for Social Sciences software 27.0, IBM, New York, NY, USA) for data analysis during the verification experiment.

## 3. Results

As a control, we compared the vertical ground reaction force during 3 s of quiet standing with and without the LPT. There was no significant difference in vertical ground reaction force between LPT and no-LPT conditions (707.50 ± 91.60 vs. 707.39 ± 91.86 N; *p* = 0.576; Cohen’s d = −0.11), indicating that the LPT did not exert a significant influence on vGRF.

### 3.1. Validity

**Figure 3:**
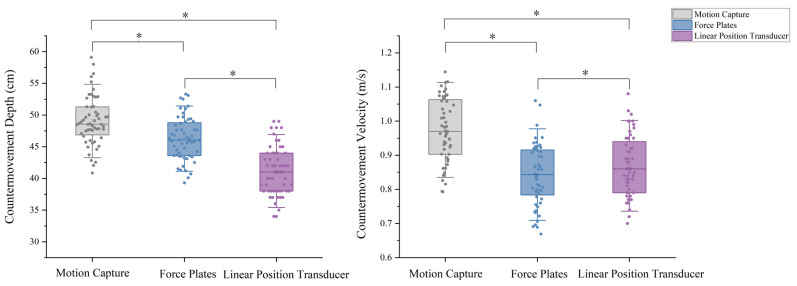
Countermovement depth, countermovement velocities (M ± SD) in the CMJ test per measuring tool. Note: * Indicates statistically significant difference, *p* < 0.05.

Results from the one-way ANOVA with repeated measures showed a statistically significant difference between the three devices for both CMD (F = 254.62; *p* < 0.001; η^2^ = 0.822) and CMV (F = 142.54; *p* < 0.001; η^2^ = 0.722) (Table 1). A post hoc comparison (Table 2 and Figure 3) showed that Motion Capture recorded significantly higher CMD than GymAware LPT (95% CI: 6.97 cm to 8.91 cm; *p* < 0.001) and Force Plates (95% CI: 2.23 cm to 3.37 cm; *p* < 0.001), while the CMV of Motion Capture also significantly higher than other devices (95% CI: 0.119 m/s to 0.143 m/s; *p* < 0.001, 95% CI: 0.083 m/s to 0.130 m/s; *p* < 0.001). The CMD and CMV of the Force Plates and Linear Position Transducer were underestimated compared to Motion Capture.

**Figure 4:**
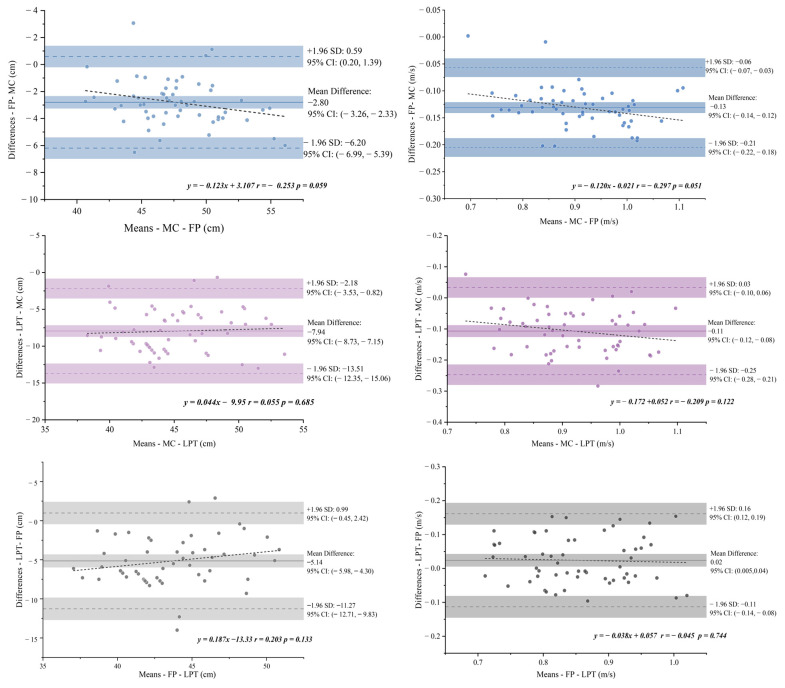
Bland–Altman plots comparing each device with motion capture (MC) for CMD (cm) and CMV (m·s^−1^). Points show difference = device − MC vs. the mean. The solid line is the mean bias; dashed lines are the 95% LoA (lower → upper); shaded bands are LoA 95% CIs. Note: The solid line represents the mean bias between methods. MC: Motion Capture. FP: Force Plates. LPT: Linear Position Transducer.

Bland–Altman plots were created to evaluate the agreement between different devices for countermovement depth and velocity (Figure 4). No proportional bias was detected for FP or LPT (*p* > 0.05). The Bland–Altman plot was consistent with the post hoc test results, which showed an underestimation of both Force Plates and Linear Position Transducer measurements compared to CMD and CMV for Motion Capture. For CMD and CMV measured by Force Plates compared with Motion Capture, the mean bias was −2.80 cm and −0.13 m/s, respectively, with limits of agreement from −6.20 cm to 0.59 cm and −0.21 m/s to −0.06 m/s. For CMD and CMV measured by Linear Position Transducer compared with Motion Capture, the mean bias was −7.94 cm and −0.11 m/s, respectively, with limits of agreement from −13.51 cm to −2.18 cm and −0.25 m/s to 0.03 m/s. For CMD and CMV measured by Force Plates compared with Linear Position Transducer, the mean bias was −5.14 cm and 0.02 m/s, respectively, with limits of agreement from −11.27 cm to 0.99 cm and −0.11 m/s to 0.16 m/s.

Relative to MC, FP showed errors of MAE = 2.97 cm and RMSE = 3.28 cm for CMD (NRMSE = 6.69%), and MAE = 0.07 m·s^−1^ and RMSE = 0.09 m·s^−1^ for CMV (NRMSE = 8.17%). Relative to MC, the LPT exhibited larger error in CMD (MAE = 7.94 cm; RMSE = 8.46 cm; NRMSE = 17.24%) but smaller error in CMV (MAE = 0.06 m·s^−1^; RMSE = 0.07 m·s^−1^; NRMSE = 7.54%), see Table 3.

**Figure 5:**
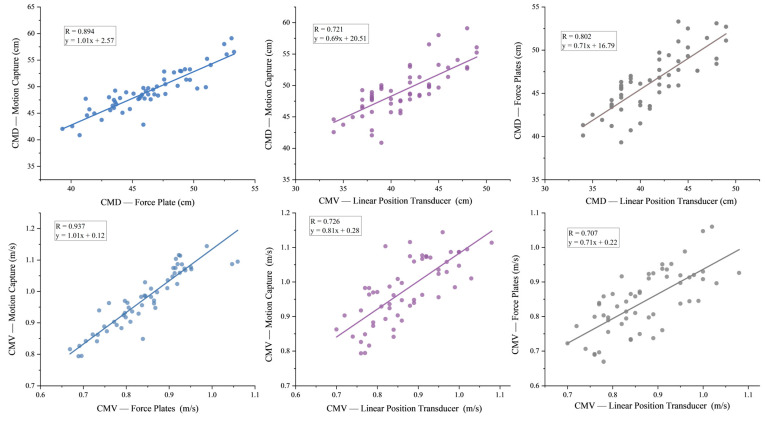
Relationship between the motion capture and linear position transducer, and between the motion capture and force plates-derived CMD and CMV. Note: CMD: countermovement depth. CMV: countermovement velocity.

The force plates (FP) and motion capture system (MC) showed very high correlations for both countermovement depth (r = 0.894, *p* < 0.001) and velocity (r = 0.937, *p* < 0.001), indicating nearly identical measurement outputs between these devices (Figure 5). The GymAware linear position transducer (LPT) had strong correlations with MC for countermovement depth (r = 0.721, *p* < 0.001) and velocity (r = 0.726, *p* < 0.001), demonstrating high correlations. Additionally, the LPT showed good correlations with FP for countermovement depth (r = 0.802, *p* < 0.001) and velocity (r = 0.707, *p* < 0.001), further supporting its reliability.

### 3.2. Test–Retest Reliability

The Force Plates and the Linear Position Transducer demonstrated good reliability for daily measurements of countermovement depth (CMD) and velocity (CMV) (ICCs > 0.70, *p* < 0.001). The linear position transducer (LPT) achieved the higher ICC values for both parameters (CMD: ICC = 0.900, 95% CI = 0.783 to 0.954, MDC = 4.73 cm, CV = 4.53%; CMV: ICC = 0.863, 95% CI = 0.704 to 0.937, MDC = 0.12 m/s, CV = 6.68%). The Force Plates (FP) demonstrated good reliability (CMD: ICC = 0.816; CMV: ICC = 0.809), with moderate limits of agreement (LOA) ranges (CMD: 95% CI = 0.603 to 0.915, MDC = 5.26 cm, CV = 4.11%; CMV: 95% CI = 0.587 to 0.912, MDC = 0.14 m/s, CV = 6.11%).

## 4. Discussion

This study aimed to establish the concurrent validity and reliability of force plates (FP) and linear position transducer (LPT) systems for measuring countermovement depth and velocity during the countermovement jump (CMJ), using motion capture (MC) as the reference standard. Our first hypothesis was not supported. Contrary to our expectations, both FP and LPT systematically underestimated CMD and CMV compared to the motion capture (MC). The motion capture system and the force plate were considered the “gold standard” for jump height during CMJ tests [15,18,38,39], and the DI method via FP could measure the JH with the least difference from the motion capture systems [21,23,40,41]. However, a certain amount of error due to integration drift is unavoidable, as demonstrated in previous studies [23,42]. The source of differences in countermovement depths and velocities measured between the MC and FP may be from integration error. Despite underestimating the measurements, the relationship between the two device measurements was very strong (r = 0.894, r = 0.937), with small mean biases (−2.80 cm; −0.13 m·s^−1^) and modest absolute and relative errors (CMD: MAE = 2.97 cm, NRMSE = 6.69%; CMV: MAE = 0.07 m·s^−1^, NRMSE = 8.17%). This information suggests that FP can be used as a valid alternative to MC for measuring CMD and CMV, but it is important to note that there are some differences between them. To comprehensively characterize jump performance, MC should be combined with FP to estimate lower-limb joint kinetics (moments, power, work) and infer neuromuscular control strategies.

The LPT underestimates the countermovement depth compared to the MC and FP, and larger absolute and relative errors (mean difference = −7.94 cm, NRMSE = 17.24%). We believe there are two reasons: (1) lower/adaptive sampling with smoothing can blunt the displacement trough and miss the true minimum, thereby underestimating depth; and (2) the waist-belt mount measures pelvis motion rather than the whole-body center of mass. This pattern contrasts with prior reports that LPTs overestimate jump height (JH) [12,13,14]. A likely reason is a method difference: when FP JH is derived from take-off velocity, it references the COM at the instant of take-off and does not include any pre–take-off heel-raise distance; by contrast, the LPT’s kinematic displacement (belt/pelvis trajectory) can include the upward motion during heel raise, thereby inflating JH relative to FP [21,23,43]. In this study, the LPT exhibited smaller error for CMV than for CMD. Although the displacement (numerator) is underestimated, the countermovement duration (denominator) may also be underestimated because the lower/adaptive sampling and coarser event timing shorten the identified window; these opposing effects partially cancel, yielding a smaller CMV error relative to MC/FP. Practically, absolute CMD from LPT should be interpreted cautiously or calibrated to MC/FP, whereas CMV appears suitable for within-athlete monitoring when force plates are unavailable.

The second hypothesis was supported, as both measurement systems demonstrated acceptable test–retest reliability for all measured countermovement parameters. The LPT showed high reliability for depth (ICC = 0.900) and velocity (ICC = 0.863), while the force plates (FP) also exhibited good reliability (depth ICC = 0.816; velocity ICC = 0.809). These results support previous research on LPTs’ measurement consistency for jump height [12,13,14], and align with established literature on vGRF-based displacement analysis using FP [15,23]. Both systems showed marginally good reliability for countermovement values (Table 4), all ICC values exceeded 0.75, and CVs were less than 10%. Therefore, countermovement depths and velocities measured by either device can be considered reliable for longitudinal monitoring. Practitioners should note significant inter-device differences in absolute COM displacement values (*p* < 0.001) and maintain strict device consistency when tracking performance changes over time.

Determining the optimal countermovement depth and velocity is crucial for maximising jump height, with research demonstrating a second-order polynomial relationship between these parameters and JH [7,22,44]. The double integration method applied to the force plate data has been the primary approach for calculating CMD and CMV in these foundational studies [5,6,7,22,44]. Consequently, the FP system remains the most widely adopted and valued tool for comprehensive CMJ assessment in both research and practice settings, prized for its established reliability, validity, and efficiency [11]. However, the FP has significant practical limitations, as it is typically large, non-portable, and requires a power source, making it less suitable for frequent field-based testing. In contrast, the LPT systems offer a portable, practical, and low-cost alternative for field use. Significantly, beyond measuring jump performance parameters (including JH and potentially optimal CMD to assess neuromuscular function), the LPT’s versatility extends to evaluating strength characteristics (e.g., bench press, squat velocity via barbell attachment) and determining training loads through velocity loss metrics [26]. While it is important to note the measurement differences between systems (particularly for countermovement parameters), this combination of portability and multi-functional assessment capability positions the LPT as a viable and practical alternative to force plates for performance monitoring in field settings.

However, several limitations of our study should be considered. First, the LPT uses proprietary commercial equipment and software, which means that its sampling rate, threshold settings, and filtering methods are unknown and cannot be synchronized with MC and FP. Second, the LPT measures pelvis motion via a waist-mounted belt, which approximates but does not replicate true whole-body COM displacement, leading to potential differences in measured values. These issues may further influence the validity of LPT measurements when compared to MC and FP. Despite these limitations, the LPT’s practical utility in field settings justifies its comparison with MC and FP, providing valuable insight into device discrepancies and helping practitioners understand the inherent differences between the devices.

## 5. Conclusions

Although the FP and LPT devices underestimated the CMD and CMV values compared to the MC criterion standard, both devices demonstrated good test–retest reliability. Since the FP showed significantly less bias and stronger correlations with the MC, in practical applications, it can serve as a reliable tool for accurate, effective, and reliable measurement of CMD/CMV. As more portable, practical, and cost-effective alternative tools, the LPTs are equally applicable to monitoring changes in countermovement strategies over the long term. However, practitioners should note that LPT measurements are subject to underestimation bias. Caution is advised when comparing CMD and CMV from various measurement systems.

## Figures and Tables

**Table 1:** Differences in countermovement depth and velocity among the three devices.

Variables	MC	FP	LPT	F	*p*	η^2^
CMD (cm)	49.07 ± 3.85	46.26 ± 3.43	41.12 ± 4.01	254.62	<0.001	0.822
CMV (m/s)	0.97 ± 0.10	0.84 ± 0.09	0.87 ± 0.09	142.54	<0.001	0.722

Note: CMD: Countermovement Depth. CMV: Countermovement Velocity. MC: Motion Capture. FP: Force Plates. LPT: Linear Position Transducer.

**Table 2:** Post-hoc Analyses of Differences in Countermovement Depth and Velocity Among the Three Devices.

Variables	MC vs. FP			MC vs. LPT			FP vs. LPT		
	*p*	95% CI		*p*	95% CI		*p*	95% CI	
CMD (cm)	<0.001	2.23	3.37	<0.001	6.97	8.91	<0.001	4.11	6.17
CMV (m/s)	<0.001	0.119	0.143	<0.001	0.083	0.130	0.038	−0.047	−0.001

Note: CMD: Countermovement Depth. CMV: Countermovement Velocity. MC: Motion Capture. FP: Force Plates. LPT: Linear Position Transducer.

**Table 3:** Agreement metrics between devices and MC. Values are mean absolute error (MAE), root-mean-square error (RMSE), and normalized RMSE (NRMSE, %).

Variables	FP			LPT		
	MAE	RMSE	NRMSE (%)	MAE	RMSE	NRMSE (%)
CMD (cm)	2.97	3.28	6.69%	7.94	8.46	17.24%
CMV (m/s)	0.07	0.09	8.17%	0.06	0.07	7.54%

**Table 4:** Test–retest reliability results for countermovement depth and velocity between testing days using different devices.

Variables	Devices	MDC	CV	ICC	95% CI	*p*
CMD (cm)	FP	5.26	4.11%	0.816	0.603–0.915	<0.001
	LPT	4.73	4.53%	0.900	0.783–0.954	<0.001
CMV (m/s)	FP	0.14	6.11%	0.809	0.587–0.912	<0.001
	LPT	0.12	6.68%	0.863	0.704–0.937	<0.001

Note: CMD: Countermovement Depth. CMV: Countermovement Velocity. FP: Force Plates. LPT: Linear Position Transducer.

## Data Availability

To protect the participants’ privacy, this study’s data will not be publicly available. At the reasonable request of the corresponding author, data supporting the results of this study can be obtained.

## Abbreviations

The following abbreviations are used in this manuscript:

CMJ	Countermovement Jump
COM	Centre of Mass
vGRF	Vertical Ground Reaction Force
CMD	Countermovement Depth
CMV	Countermovement Velocity
MC	Motion Capture
FP	Force Plates
LPT	Linear Position Transducer
DI	Double Integration Method
JH	Jump Height
ICC	Intraclass Correlation Coefficient

## Appendix A

**Table A1:** List of markers and corresponding locations.

Marker Name	Location/Description
H1-H3	Head (Frontal/Occipital/Temporal, 3 points)
C7	Spinous process of the 7th cervical vertebra
T10	Spinous process of the 10th thoracic vertebra
L/R_SAE	Scapula—Acromial Edge
L/R_SIA	Scapula—Inferior Angle
L/R_HUM	Humeral wand (Left)
L/R_HLE	Humerus—Lateral Epicondyle
L/R_HME	Humerus—Medial Epicondyle
L/R_FAR	Forearm wand
L/R_USP	Ulna—Styloid Process
L/R_RSP	Radius—Styloid Process
L/R_HM2	Dorsal aspect of the second metacarpal head
L/R_IAS	Anterior Superior Iliac Spine
L/R_IPS	Posterior Superior Iliac Spine
L/R_GT	Greater Trochanter
L/R_TH1-4	Thigh cluster—4 markers
L/R_FLE	Femur—Lateral Epicondyle
L/R_FME	Femur—Medial Epicondyle
L/R_SK1-4	Shank cluster—4 markers
L/R_FAL	Lateral prominence of the lateral malleolus
L/R_TAM	Medial prominence of the medial malleolus
L/R_FM1	Dorsal margin of the first metatarsal head
L/R_FM2	Dorsal aspect of the second metatarsal head
L/R_FM5	Dorsal margin of the fifth metatarsal head
L/R_TOE	Toe Tip
L/R_FCC	Posterior aspect of the calcaneus
